# Infections in early life and risk of childhood ALL

**DOI:** 10.1038/sj.bjc.6604950

**Published:** 2009-03-03

**Authors:** M Greaves, P A Buffler

**Affiliations:** 1 Section of Haemato-Oncology, The Institute of Cancer Research, London, UK; 2 Division of Epidemiology, School of Public Health, University of California, Berkeley, CA, USA

**Sir,**

Cardwell *et al* (2008) report that a survey of GP records, detailing recorded infections in the first year of life, finds no evidence to support the hypothesis by Greaves (2006) that deficient infectious exposure in infancy may be a risk factor for developing the common form of childhood acute lymphoblastic leukaemia (ALL). This study therefore confirms the earlier report by Roman *et al* (2007). These data indeed provide no support for the hypothesis. However, the authors’ underlying premise bears examination. Nowhere in the ‘Greaves’ (‘delayed infection’) hypothesis does it state or predict that the relevant ‘protective’, infectious exposures in infancy or early childhood will necessarily elicit overt symptoms or pathology that prompt GP visitations. It is perfectly plausible that only particular, albeit common, infections are, for historical/evolutionary reasons (Greaves, 2006), competent to appropriately modulate the neonatal immune system network, and these could be essentially innocuous or ‘invisible’ infections as in the parallel ‘Old Friends’ hypothesis proposed by Rook (2007) for risk of allergies. We suspect that a large proportion of infections in infancy and early childhood are asymptomatic. Has anyone done a careful study of this? A biological measure might be more relevant for an evaluation of the hypothesis than querying GP records. The authors (Cardwell *et al*, 2008) also ignore a conflict of data that requires resolution. The lack of protection afforded by infectious episodes recorded in GP records is at odds with a large and growing body of data from case–control studies that indicate that attendance at playgroups in infancy is protective for childhood ALL. This latter conclusion is a consistent finding from the largest studies designed to address the question (Gilham *et al*, 2005; Ma *et al*, 2005; Kamper-Jørgensen *et al*, 2007). Day care attendance is an accepted surrogate indicator for all types of infections transmitted through personal contact and is currently the best test of the ‘Greaves’ hypothesis as it makes no assumptions about microbial species or associated pathology/symptoms. The authors of the recent reports (Roman *et al*, 2007; Cardwell *et al*, 2008) are correct to conclude that their data provide no support for the ‘delayed infection’ hypothesis, but neither do they negate it.
